# Origin of Life and Birth of *Life* — An Open Access Journal

**DOI:** 10.3390/life1010001

**Published:** 2011-08-23

**Authors:** Shu-Kun Lin

**Affiliations:** MDPI AG, Postfach, CH-4005 Basel, Switzerland; E-Mail: lin@mdpi.com; Website: http://www.mdpi.org/lin/

Our publishing company MDPI (Multidisciplinary Digital Publishing Institute) planned to launch this journal *Life* (ISSN 2075-1729) since June 2009. Life science as a topic covers a very broad area. We decided to focus the scope of this new journal on the origin of life and the evolution of biosystems such as molecular evolution. Of course any fundamental theoretical topics and experimental discoveries in biology, biochemistry and biophysics will be welcomed also.

Life science is a typical multidisciplinary research field that chemists like me can also participate in. Specifically, the open question of the origin of life can be attacked by scientists, including many curious chemists, physicists and biologists. In another MDPI journal the *International Journal of Molecular Sciences*, 23 papers guest edited by the chemistry professor Marie-Paule Bassez were published in the special issue “Origin of Life” [1]. A second edition of this successful special issue “Origin of Life 2011” will be published in 2011, also in the *International Journal of Molecular Sciences*, and has been guest edited by Prof. Dr. Jack Green and Prof. Dr. Robert Root-Bernstein [2].

Many authors believe the well-known processes of molecular recognition (such as enzymatic processes), protein folding, and molecular self-assembly are driven by energy minimization while I think entropy increase is the main or the exclusive driving force [3,4,5]. Of course these processes should be part of the origin of life. As the publisher of *Life* (ISSN 2075-1729), I will be pleased to run this journal as a good venue to publish high quality research results and to promote the progress of life science. All kinds of different opinions, theories and experimental findings are welcomed for consideration and publication.

The fundamental problems in science interest a large readership. As an open access journal, *Life* (ISSN 2075-1729) will be an ideal scholarly avenue to bring such important research achievements to a wide audience.

Enjoy publishing with us.
